# TKFIM: Top-K frequent itemset mining technique based on equivalence classes

**DOI:** 10.7717/peerj-cs.385

**Published:** 2021-03-08

**Authors:** Saood Iqbal, Abdul Shahid, Muhammad Roman, Zahid Khan, Shaha Al-Otaibi, Lisu Yu

**Affiliations:** 1 Institute of Computing, Kohat University of Science & Technology, Kohat, Kohat, KPK, Pakistan; 2 Robotics and Internet of Things Lab, Prince Sultan University, Riyadh, Saudi Arabia; 3 Information Systems Department, College of Computer and Information Sciences, Princess Nourah Bint Abdulrahman University, Riyadh, Saudi Arabia; 4 School of Information Engineering, Nanchang University, Jiangxi, China; 5 State Key Laboratory of Computer Architecture, Institute of Computing Technology, Chinese Academy of Sciences, Beijing, China

**Keywords:** Frequent Itemsets, Support Threshold, Algorithm Analysis, Top-k Frequent Itemsets, Artifical Intelligence

## Abstract

Frequently used items mining is a significant subject of data mining studies. In the last ten years, due to innovative development, the quantity of data has grown exponentially. For frequent Itemset (FIs) mining applications, it imposes new challenges. Misconceived information may be found in recent algorithms, including both threshold and size based algorithms. Threshold value plays a central role in generating frequent itemsets from the given dataset. Selecting a support threshold value is very complicated for those unaware of the dataset’s characteristics. The performance of algorithms for finding FIs without the support threshold is, however, deficient due to heavy computation. Therefore, we have proposed a method to discover FIs without the support threshold, called Top-k frequent itemsets mining (TKFIM). It uses class equivalence and set-theory concepts for mining FIs. The proposed procedure does not miss any FIs; thus, accurate frequent patterns are mined. Furthermore, the results are compared with state-of-the-art techniques such as Top-k miner and Build Once and Mine Once (BOMO). It is found that the proposed TKFIM has outperformed the results of these approaches in terms of execution and performance, achieving 92.70, 35.87, 28.53, and 81.27 percent gain on Top-k miner using Chess, Mushroom, and Connect and T1014D100K datasets, respectively. Similarly, it has achieved a performance gain of 97.14, 100, 78.10, 99.70 percent on BOMO using Chess, Mushroom, Connect, and T1014D100K datasets, respectively. Therefore, it is argued that the proposed procedure may be adopted on a large dataset for better performance.

## Introduction

Finding FIs is one of the leading research problems used in many critical data mining tasks like classification (Nguyen et al., 2012), clustering (Wang et al., 2002), sequential patterns (Fournier-Viger et al., 2017), and association rule mining (ARM) (Agrawal, Imielinski & Swami, 1993). Besides this, other various applications such as multitask based association rule mining (Taser, Birant & Birant, 2020), high utility pattern mining (Krishnamoorthy, 2019), Top-k pattern mining (Nguyen et al., 2018), and frequent weighted mining (Nam et al., 2020b). FIs mining methods find the set of items that frequently occurs in a given set of transactions. It shows the association rules which define how an itemset in a transaction dataset depends on another itemset’s behavior. The first algorithm used for computing and finding association among FIs is known as Apriori (Agrawal, Imielinski & Swami, 1993; Agrawal & Srikant, 1994). The Apriori algorithm generates a large number of candidate itemset. It also performs multiple scanning of the transaction table for finding frequent itemsets that result in overhead on input and output devices. For large database systems, the I/O overhead becomes more demanding for large memory for storing the data. Later on, Zaki & Gouda (2003) proposed the dEclat, a diffset algorithm. It employs a vertical representation of the database. The fundamental concept of a diffset algorithm is that a particular set of transaction IDs (tidsets) can be used to measure the support of itemsets. The main serious demerit of the Eclat approach is the size of tidsets, which affect the processing time and are costly to store in memory. Another algorithm that influenced most of the work in this area is Frequent Pattern (FP) growth (Han, Pei & Yin, 2000). The FP-Growth method performs at least two scans. First, process frequent patterns of length-1 and count their support value. Afterward, the items are sorted in decreasing order of their support. These methods are referred to as conventional methods.

The conventional FIs methods are based on the minimum support threshold (Fournier-Viger et al., 2017; Agrawal & Srikant, 1994). In a transaction table, the minimum supported value, also known as minsup, specifies the minimum frequency of an element in a collection. All the itemsets whose frequency exceeds or is equal to the threshold value are known as FIs. However, it is a difficult task to find a reasonable value for a threshold. For example, if the threshold value is maintained too low, too many FIs can be created, and the necessary patterns can hardly be found among the massive collection of produced patterns.

Similarly, if the threshold value is set too high, it will generate too few FIs, in which we may miss some crucial patterns in the transaction table. The selection of the threshold value also affects the field of the search and the resulting space (Huynh-Thi-Le et al., 2015). Thus another set of methods emerges; they are referred to as Top-k procedures. It is the procedure to find out itemsets of highest support to the k support among all the existing FIs. It refers to the user’s choice of frequent itemsets in the dataset. User choice allows the user to find Top-most frequent itemsets. Top-most early frequent itemsets procedure finds the Top-most frequent itemsets by repeating the mining process until the desired result is obtained. These approaches generally require more execution time and produce ample result-space, resulting in redundant patterns in the transaction table. N-most is a Top-most frequent itemset mining technique that processes the top N impressive results without specifying any user-parameter (Fu, Kwong & Tang, 2000). It makes use of the Apriori candidate creation procedure and test strategy. It first computes the largest itemset and then compares the support of candidate itemsets, recursively. In every iteration, it updates the support threshold of itemsets. The process continues until the user specified bound on the itemset size. The Top-most mining technique is the Top-k frequent itemsets mining. Unlike N-most frequent itemsets mining procedure, Top-k finds itemsets of highest support without specifying the itemset size. Top-k frequent itemsets mining may be divided into support threshold and without support threshold-based mining algorithms. These algorithms may also be categorized into algorithms based on Apriori and FP-growth (Agrawal, Imielinski & Swami, 1993; Han, Pei & Yin, 2000). The Top-k algorithms (based on Apriori) build 1-itemset and then attach to 2-itemset and so on. In the end, the results are compared with a user-specified threshold value.

Top-k algorithms based on FP-growth use FP-tree for pattern mining. It splits the transactions to reduce the search-space. The critical advantage of FP based algorithms is that they use a tree structure of a transaction database. The disadvantage of using a tree is its difficulty in being stored in memory, and its building procedure is costly. Han et al. (2002) proposed TFP (Top-k frequent closed itemsets mining algorithm), which uses FP-tree without the user’s support threshold. The algorithm constructs the FP-tree based on the primary support threshold starting with zero. It prunes smaller transactions using itemsets length constraints. Mining is performed on the final pruned FP-tree. The algorithms discussed above need to scan the transaction table multiple times to build the tree. They also consume large search-space and uses expensive strategies to improve performance. Details of these methods are discussed in the related work section.

### Research gap

However, summarizing the limitation of the previous studies that are (1) The absence of user-specified support threshold parameter can affect the performance of the FIs mining algorithms, (2) the generation of the exponential number of itemsets and support counting is difficult to handle in Top-k FIs mining techniques, and finally, (3) effectively trimming those transactions that are not useful for the next level, which increases the processing time and degrades performance are the main challenging areas to be handled.

To overcome these limitations, we proposed Top-k Frequent Itemsets Mining (TKFIM) algorithm finding FIs without a user-specified support threshold. The working of TKFIM is based on concept equivalence classes of set theory. Each class is an independent group of itemsets. Further, it uses diffset to count the support of the itemsets. The proposed procedure applies to a vertical database structure consisting of transaction IDs (tids) followed by items. Our algorithm adopts a class-based search strategy to quickly find itemsets of highest support, and it mines the candidates of the current class-based on the joining process. If the support of an itemset is less than the least support in the Top-k list, then the current class terminates the generation of candidate itemsets. The next class joining process is then applied accordingly. The process is repeated until no frequent or candidate itemsets are found. Finally, the results of the proposed system are compared with BOMO and Top-k miner on multiple datasets.

The contributions of this paper are listed as follows:

1. It presents FIs based Top-k algorithm that reduces the number of scans and decreases the run time.
2. It finds all frequent FIs and IFIs, and do not miss any pattern.
3. This research provides a comprehensive review of the existing literature in the field of frequent itemset mining.
4. Based on the critical analysis, this paper highlights the limitations of the state-of-the-art research studies used for FIs mining.
5. The pruning strategy is used in this paper to reduce the number of candidate frequent itemsets.
6. Afterwards, a novel approach (i.e., TKFIM) is proposed, designed, implements based on equivalence classes and diffset theory.
7. The experimental results show that TKFIM has a significant advantage over the existing algorithms.

Finally, TKFIM results are compared with BOMO and Top-k miner techniques. These algorithms are evaluated on five different datasets, i.e., Chess, Mushroom, Connect, and Synthetic dataset. Further, the performance gains on each dataset are recorded. In the first experiment, on the Chess dataset, the average performance gain of 97.14% and 92.70% was achieved compared to BOMO and Top-k miner, respectively. Similarly, on the Mushroom dataset, more than 100% and 35.87% performance gain was achieved concerning BOMO and Top-k miner. On the third dataset, i.e., Connect 78.10% and 28.53% performance gain was delivered compared to BOMO and Top-k miner, respectively. In the final experiment on the Synthetic dataset (T1014D100K), the average performance gain of 99.70% and 81.27%was recorded for BOMO and Top-k miner.

## Related Work

In the area of Frequent Itemset Mining, the very first algorithm, i.e., Apriori, was proposed by Agrawal, Imielinski & Swami (1993). This algorithm uses a bottom-up search method to process the extraction of frequent itemsets. It handles all itemsets in k-steps where k represents the size of a given database. In the first step, all the frequent 1-itemsets are generated. In the second step, all the frequent 1-itemset are joined to compute 2-itemsets, compare their support with the given specified minsup. All the frequent 2-itemsets are processed for the subsequent 3-itemsets. The process continues until no itemsets can be found. Another classical algorithm referred to as Eclat was proposed by Zaki (2000). The transaction database and minsup are used as the input for this algorithm. It counts the support of itemsets using tids, which is the length of the itemset.

Eclat algorithm is more efficient than those algorithms which repeatedly scans the database, but it requires more memory to store the tidsets. dEclat algorithm is a variation of the Eclat algorithm implemented using a structure called diffsets (Zaki & Gouda, 2003) rather than tidsets. FP-growth is proposed by Han, Jiawei, and Pei, Jian in 2000 for finding frequent itemsets without candidate generation (Han, Pei & Yin, 2000). It uses FP-tree for computing frequent itemsets by scanning it at least twice. In the first scan, it processes frequent patterns of length-1 and counts their support value. In the second scan, the items are sorted in decreasing order of their support. It splits the database to build multiple conditional FP-trees, which considerably reduces the search-space. FP-growth works better than Apriori because the data in the memory is a tree structure form. All the conventional algorithms involve an enormous search-space, and it may expand exponentially. The database also needs to be searched regularly, which requires a lot of runtimes. However, since the threshold parameters for these algorithms are required, selecting the threshold value remains a difficult task. If the threshold value remains very high, too many items will be produced.

On the other hand, it can lead to too few frequent items when support is too high. Top-most FIs itemset mining algorithms are proposed to solve this issue of traditional FIs mining methods,

### Top-most FIS methods

Top-most FIs refers to the user choice of FIs in the dataset. User choice allows the user to find Top-most FIs. Top-most early FIs procedure finds Top-most frequent itemsets by repeating the mining process until the desired result is obtained. The researcher found two significant problems in early Top-most FIs procedures. First, it takes much more execution time to find the result. Secondly, it produces large search-space and result-space. Recently, a novel scheme of Top-most FIs mining called N-most interesting frequent itemsets has been projected (Fu, Kwong & Tang, 2000). It processes the Top-N impressive results without specifying any user parameter. It makes use of the Apriori candidate creation procedure and test strategy. It first computes the largest itemset in the dataset. The N-largest itemsets mining compares the support of the candidate itemsets recursively and updates the support threshold at the end of the iteration. The process is iterated and stops at the user-specified bound on the itemset size. The Top-most FIs mining is divided into two different sets of mining Processes, including N-most and Top-k itemsets. The details are discussed in the following sections.

### N-most interesting FIS procedures

It combines the N-most interesting frequent k-itemsets for every K where 1<= K <=m and K is the user-supplied maximum size of the itemsets to be mined. This mining process comes from the Itemset-iLoop and Itemset-Loop algorithms (Fu, Kwong & Tang, 2000). The Itemset-Loop is the first technique proposed in N-most interesting frequent itemsets mining category. Its method of candidate creation is similar to the Apriori candidate process. Itemset-loop algorithm first computes the set of potential 1-itemset, and then the new potential 2-itemsets are rooted from 1-itemsets. In the next iteration, new potential 3-itemsets are produced from a 2-itemset. The process is iterated and ends at the user-specified hop on the itemset sized. Hence it requires loops back in the kth iteration to generate N-most exciting itemsets. The idea of the Itemset-iLoop method is similar to that of Itemset-Loop, except it goes back first to check k -1 itemsets.

The underlying principle for both methods is that if a K-itemset is not frequent, all its subsets are rare. For mining N-most intriguing itemsets, this Apriori standard does not apply. Cheung & Fu (2004) have introduced a technique based on the principle of Build Once and Mine Once (BOMO) to address the drawbacks of the most interesting items in mining. This procedure is based on the free parameter for N-most exciting items. The BOMO is a technique based on FP-growth that uses the inherent characteristics of the compact pattern-tree structure. It works without candidate generation to improve results in frequent itemsets mining problems. Being an FP-growth-based approach, BOMO suffers from common demerits, such as scanning the database multiple times to evaluate itemsets’ support. FP-tree structure is time-consuming, and visiting nodes to evaluate the support of itemsets is very in-efficient. Consequently, the size of the database is immense. Thus it may not be possible to store it in the main memory. As a result, the result set will cause the failure of the mining operation.

### TOP-K FIs mining methods

The Top-k FIs mining methods can be categorized as support threshold and non-threshold-based algorithms. Most frequent pattern mining algorithms depend on the right minimum support threshold parameter. However, as already discussed, it is very tricky in practice to determine the right value for this parameter. Therefore another category of Top-k frequent itemsets algorithms is proposed, which are threshold-free. The Top-k FIs mining techniques are also classified as Apriori and FP-based algorithms.

The Apriori based techniques generate FIs of 1-itemsets, and then produced 2-itemset by joining them, and similarly 3-itemsets, and so on. On the other hand, FP-growth based Top-k FIs techniques make use of FP-tree for frequent mining patterns. It divides the transactions to reduce the scope of the search. The main advantage of FP-growth based algorithms is that the tree data structure is an unstructured form of the transaction database. These types of algorithms cannot be stored in memory and, therefore, costly to build. However, vertical format based techniques are more intuitive and straightforward as compared to horizontal format developing approaches.

Top-k Frequent closed itemsets, the algorithm called TFP without the minimum threshold using the FP tree, have been suggested by Han et al. (2002). The algorithm begins with the FP-tree, the primary threshold being set at zero. The smaller transaction, i.e., a transaction with length <minimum length, is pruned during the tree’s construction. After FP-tree construction, it uses a closed node count and a descendant sum method to prune the relatively unusual patterns by increasing the support threshold. In order to accelerate the process, the TFP algorithm uses FP-tree accessing strategies such as bottom-up and top-down.

Mining is performed on the final pruned FP-tree. On the other hand, the TFP algorithm has many demerits. For the FP-tree, the database must be scanned twice. The TFP is an FP-growth-inspired method and uses two parameters. This algorithm consumes a large search-space and uses expensive strategies to improve performance. Amphawan, Lenca & Surarerks (2009) have focused on finding Top-k periodic-frequent patterns. It initially considers the highest support patterns and then combines candidates to form the Top-k periodic-frequent patterns list. Pietracaprina & Vandin (2007) suggested that Top-k Miner find Top-k frequently closed unlimited items. The algorithm starts primarily with approximate minimum support having heuristics similar to those references in the above Top-k closed frequent itemsets mining procedures. This procedure has dynamically raised the support threshold with no need to restart computation. It uses the priority queue to stop comparing the support of closed itemsets. Further, it adopts the best-first search to produce the first highest support closed itemsets. At the point when all the closed itemset support is processed, it terminates the main loop. At this point, k is assigned a new value, and the loop repeats to generate the highest support closed itemsets.

A naive approach called the Combination Reducing Method (CRM) is recently proposed by Pyun & Yun (2014). This algorithm reduces time and saves memory by applying the composite pattern concept. The CRM is FP-growth based algorithm that constructs a conditional FP-tree.

The algorithm starts with an initial support threshold of zero while building the FP-tree from the dataset. Following this, the algorithm constructs a global FP. During the development of the FP-tree, a header table is generated simultaneously. The sequence of selecting the prefix is different from FP-growth. The prefix is the center item in the header table that is quite suitable for the mining of Top-k frequent patterns. It can raise the threshold effectively and process patterns quickly. CRM does not consider the pattern length, so the Top-k patterns are placed without length constraints into the Top-k list. The current Top-k Composite pattern list and the recovery phase are used to create Top-k patterns. On the other hand, the CRM algorithm has numerous disadvantages. To build the FP-tree, it still has to scan the database twice. CRM is an FP growth inspired approach and uses two parameters. This algorithm requires a large search-space and is expensive using the recovery phase to improve the performance. The Top-k list includes many redundant composite patterns when the value of k is put as a large.

Pyun & Yun (2014) also developed a combination reduction method for N-itemsets (CRMN) followed itemset length constraints. CRMN scans the dataset at least two times, and initial support is set to zero. In the mining procedure, CRMN first constructs FP global-tree. It subsequently mines Top-k patterns from FP-global-tree. During this process, the composite patterns are grouped into k-patterns for each length in the Top-k list if the present conditional FP tree has one-path. If the tree has a multipath, the algorithm will detect frequent patterns and insert them into the Top-k list according to its lengths.

Salam & Khayal (2012) proposed Top-most and Top-k methods based on the association graph structure. A Symmetric matrix stores the entry of the graph as a data structure. The algorithm scans the database once and starts with the FP tree with the initial support threshold of zero during the construction of the All-path Source to Destination (ASD)-tree (Salam & Khayal, 2012). This method mines search-space and construct an ASD tree simultaneously. It suffers from serious demerit that it computes all 2-itemsets and scans each transaction to build an association ratio graph. It computes edge values simultaneously to detect a maximum cycle. The other drawback in this approach is that it identifies those itemsets as Top-k itemsets, but originally itemset has low dataset occurrence.

Recently, Saif-Ur-Rehman et al. (2016) suggested a Top-k miner approach to find FIs. They scan the database once and find a supporting threshold higher than zero for all the frequent 2-itemsets. They use Candidate itemsets Search Tree (CIS-tree) (Saif-Ur-Rehman et al., 2016) to mine the desired number of Top-k FIs of highest support.

The technique suffers from various limitations, such as it computes 2-itemsets before constructing the CIS-tree, which is expensive to build as it consumes much memory. This algorithm has reduced the search-space but has increased the runtime of the mining process. Furthermore, the frequent itemsets joining procedure for the construction of CIS-tree is a time-consuming process. As discussed above, several frequent itemset mining practices were proposed, but they all face a challenge to reduce the time needed to calculate and reduce the space needed to perform the algorithm to mine all the frequent patterns required. The summary of Top-k FIs mining algorithms’ strengths and weaknesses are shown in Table 1. It suggests that Top-k frequent mining algorithms are needed to be further improved to produce efficient output.

## Proposed Methodology

The objective of this paper is to present algorithms that can find Top-k frequent itemsets without using the *minsup* parameter. The proposed algorithm begins by requesting that the user provide a “k” value, i.e., how many FIs does he required? The system proposed initially generates a frequent itemset of size one by scanning the database for the first and last time. Then the system uses equivalence classes to create the next frequent itemset level. The process repeats; initialize the item k at the next level to the lowest support until no frequent items or items can be found.

The proposed work has two significant advantages: it does not require a support threshold, and secondly, the database is scanned once to generate FIs. The detailed working of the proposed technique is discussed in this section. Before going into exact steps, a few definitions are essential to understand.

**Def-1: Frequent Itemsets:** In a given database D, an itemset X is referred to as Frequent Itemsets if its support is greater or even equal to the support of k itemset in a set of items I, where I is the set of items in the decreasing support order the support value is calculated from the user-supplied value of k (Saif-Ur-Rehman et al., 2016).

**Table 1:** Summary of the Top-K Frequent Itemsets Mining Algorithms.

**S.No**	**Algorithm**	**Purpose of algorithm**	**Storage**	**Strengths**	**Limitations**
**1**	Top k closed frequent patterns (TFP) (Han et al., 2002)	Generate the top k closed patterns for specified value of k	Array	Without support threshold Limit on candidates’ *FIs*. Itemsets mining method without candidates production	• Scan the database at least 2- times.
					• It misses certain important patterns
					• Due to Itemsets length restriction.
					• Represent the only Itemset of higher support in the Top-k while other itemsets of similar support are not considered as top- k itemsets.
**2**	Top-N (Fu, Kwong & Tang, 2000)	Generate the topmost patterns for specified value of N	Array	Without support threshold FIs method	• Approach multiple scans
					• Set two parameters.
					• Apriori Based
					• Forced to reduce Search-Space
					• Huge Search Space
**3**	CRMN (Pyun & Yun, 2014)	To generate Top k Patterns with Combinations reducing method for N- itemsets.	FP- tree	Without support threshold. Itemsets mining method without candidates’ production.	• More than 1- inputs parameters
					• STP Based Approach
					• Forced to reduce Search-Space
					• Scan DB multiple times
					• Required high computation time
**4**	CRM (Pyun & Yun, 2014)	To generate Top k patterns with Combinations reducing method for N- itemsets.	FP- tree	Without support threshold. Itemsets mining method without candidates’ production.	• Search-Space focused
					• Huge Search Space
					• Scan DB multiple times
					• Heavy computation time required
**5**	Top-k maximal FIs without support threshold (Salam & Khayal, 2012)	To find Top-most FIs based on association ratio graph structure.	Graph	Without support threshold.	• It computes all 2-itemsets and scans each transaction to build all path sources to destination tree (ASD Tree) simultaneously.
					• It identifies those itemsets as Top-k itemsets originally itemset has low occurrence in the dataset.
**6**	Top-k Miner (Saif-Ur-Rehman et al., 2016)	To find Top-k FIs without support threshold using CIS Tree.	CIS Tree	Without support threshold. Reduced the Search Space	• High computation time required high memory Consumptions.
					• The FIs joining procedure is a time consuming process until the desired result is obtained.
**7**	TKFIM [Proposed]	Top-k frequent itemsets Mining without support threshold based on Equivalence classes	Lookup List	Reduce search space and run time	• The Top-k-FIs mining first generates the entire 1-itemsets with support from the given dataset which consumes huge memory at first level.
					• Search space focused Technique

**Def-2: Identical Frequent Itemsets:** In the given dataset D, Identical Frequent Itemset (IFIs) are those frequent itemset S = X1, X2…Xm has the same support as of set S where 1<=m<=I and S ⊆ I (Saif-Ur-Rehman et al., 2016).

**Def-3: Top-k Frequent Itemsets:** A set of all IFIs in S= {X1, X2,…..Xm} is called Top-k frequent itemset if it includes frequent itemset of the highest support to the k th support itemset in I, where I is a set of all identical frequent itemsets (Yildirim, Birant & Alpyildiz, 2017).

### Top-k List Structure

In this sub-section, we describe list (Amphawan, Lenca & Surarerks, 2009) is an efficient data structure uses in our method to accommodate candidate itemsets. The list is created dynamically based on the itemset to be generated, which holds all possible k itemsets based on users supplied value of k.

**Figure 1:**
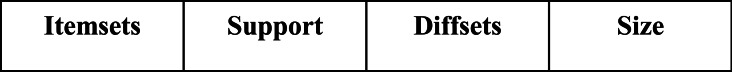
Structure of Top-k list.

The items in the list are sorted in support descending order. Using a linked list in the suggested algorithm effectively reduces processing time and retrieves the itemsets from memory faster than an expensive I/O operation. The proposed TKFIM algorithm uses vertical format datasets to generate frequent itemsets. List contains the set of nodes at different levels. The 1-itemsets, 2-itemsets, or more are generated from the vertical dataset. The Top-k list does not store any non-frequent itemset. The Top-k list structure of a node is given in Fig. 1:

Where the Top-k list structure of a node having first field is used to store itemset, the second is the support field identified the number of times the itemset occurs in the dataset. Diffset is the third field which stores the difference set of transaction id of transactions. The last field’s size points to the level of an itemset.

### Detailed working of the algorithm

It is a difficult task to find Top-k frequent itemset in large databases. This section presents the evolution of a new Top-k mining algorithm to calculate Top-k all IFIs without the support threshold. The proposed procedure applies to a vertical database structure that consists of transaction IDs followed by Items. Our technique uses a class-based search strategy for searching itemsets with the highest values of support, and it mines the candidates of the current class-based on the join process. The candidate itemsets generation for the current class will be avoided If an itemset has less support than the least support in the Top-k. The next class membership process is then applied accordingly and repeated until no frequent items or candidate items have been identified.

**Table:** 

Algorithm 1	
Top-k FIs Mining Algorithm	
1:	Scan D to generate 1-itemset with support count
2:	Create top-k list and 1-itemsset is initialized
3:	Order Top-k list in descending order of the support count
4:	Smallest *k* assign to the least support k^th^ itemset in the Top-k list
5:	Generate Candidate-Itemsets with support count using diffset
6:	Create a list Top-k
7:	**while***k* <*l en l i st***do**
8:	*k* 1 + +
9:	**while***k* 1 <*l en l i st***do**
10:	**if***pr e f i x A* == *pr e f i x B***then**
11:	*I tems*← [*I tem A* , *I tem B* ]
12:	*ke y*←*pr e f i x A* + *I tems*
13:	*diffset*←*t* (*Item A*) −*t* (*ItemB*)
14:	*support*←*t* (*Item A*) –*diffset*
15:	**end if**
16:	Create an itemset entry and insert it into the Top-k list
17:	**if***support* >= *smallestk***then**
18:	*I temset* [*key*] ←*support*
19:	**else if***support* <*smallestk***then**
20:	break
21:	**end if**
22:	**end while**
23:	*k* 1 + +
24:	*k* + +
25:	**end while**
26:	Repeat step 3, 4 and 5 until no candidate itemsets can be found
27:	Return Top-k list of *FIs*

The pseudocode of our proposed methodology is given in Algorithm 1, and the details of each step are described below:

1. Scan the Database and transform it into a vertical format.
2. Sort all items in descending support order.
3. Initializing Top-k list to 1-Itemset, where k is the user-provided number of a frequent itemset.
4. Computing 2-itemsets from 1-itemset using diffset till the least support itemset in Top-k list using equivalence class concept.
5. The process continues until the lowest support of an item on the next stage and no frequent items or candidates can be found.
6. Then, the Top-k list is returned.

**Lemma 1:**

In the frequent itemsets mining, the support value of the itemset is used to apply the anti-monotone property.

### Proof:

Suppose that there are n transactions in the database, and data structures are constructed. There are patterns P in D with m length and pattern P′ in D, which is supper pattern of P, with m+1 length. In the worst case, both P and P′ are included in T (*k* <  = *n*). The anti-monotonic property means that if any P is an invalid pattern, the supper pattern P′ of P is also an invalid pattern, which must be satisfied in this method. However, in the general case, the set of transactions containing P′ is a subset of the set of transactions containing P. Thus, Sup(P′ ) <= Sup(P) is always established. If the P is an invalid pattern, sup(P′) <= sup(P) <= misupp is valid. As a result, the itemset count value that satisfies the anti-monotone property (Nam et al., 2020a).

### Running example of TKFIM algorithm

The algorithm is expressed by using database *D,* as shown in Table 2. It contains ten transactions having six different items. Consider the number of required results specified by the user *k* is four and six. Our task is to find the Top-k itemsets with the highest support from the given transaction dataset. Table 3 illustrates the generated Top-2, Top- 4 and Top-6 frequent itemsets from the given transaction database *D*. The first column in transaction databases represents the tids of itemsets, whereas the second column describes the itemsets.

### Step 1: Generating 1-itemset by scanning the Transaction database

The format of the input dataset is of considerable importance in most of the frequent itemsets mining algorithm. There are two types of dataset representations. One is a horizontal data format, and the other is a vertical data format. In a horizontal data format, a transaction database is similar to a list/array of transactions. Each of the TID contains the itemsets. The horizontal transaction database format is given in Table 4.

**Table 2:** Transaction Database.

TID	Items
1	A,B,D,F
2	A,B,F
3	A,D,F
4	B,C,D,E,F
5	B,C,D,E,F
6	A,B,F
7	A,B,D,F
8	A,B,F
9	A,B,C,D,F
10	A,B,C,E,F

In a vertical data format, each record represents a transaction. This transaction contains itemsets followed by a distinct transaction identifier. The vertical transaction database format is shown in Table 5.

**Table 3:** Generated Top-2, Top-4 and Top-6 Itemsets.

Top-k FIs	Top-2 FIs	Top-4 FIs	Top-6 FIs
1	F:10	F:10	F:10
2	B:9, BF:9	B:9, FB:9	B:9, FB:9
3		A:8, FA:8	A:8, FA:8
4		BA:7	BA:7
5			D:6, FD:6
6			BD=5, FBD=5

Most of the previous work adopts horizontal dataset representation. Our algorithm has used the vertical layout of the dataset because the horizontal data format has some drawbacks. We first transform the input horizontal transaction dataset into a vertical format with only frequent itemsets and their corresponding transaction IDs. This generates the k-itemsets of size one, as shown in Table 6.

**Table 4:** Horizontal database format.

TID	Items
1	A,B,D,F
2	A,B,F
3	A,D,F
4	B,C,D,E,F
5	B,C,D,E,F
6	A,B,F
7	A,B,D,F
8	A,B,F
9	A,B,C,D,F
10	A,B,C,E,F

### Step 2: Sort all items in descending support order

**Figure 2:**
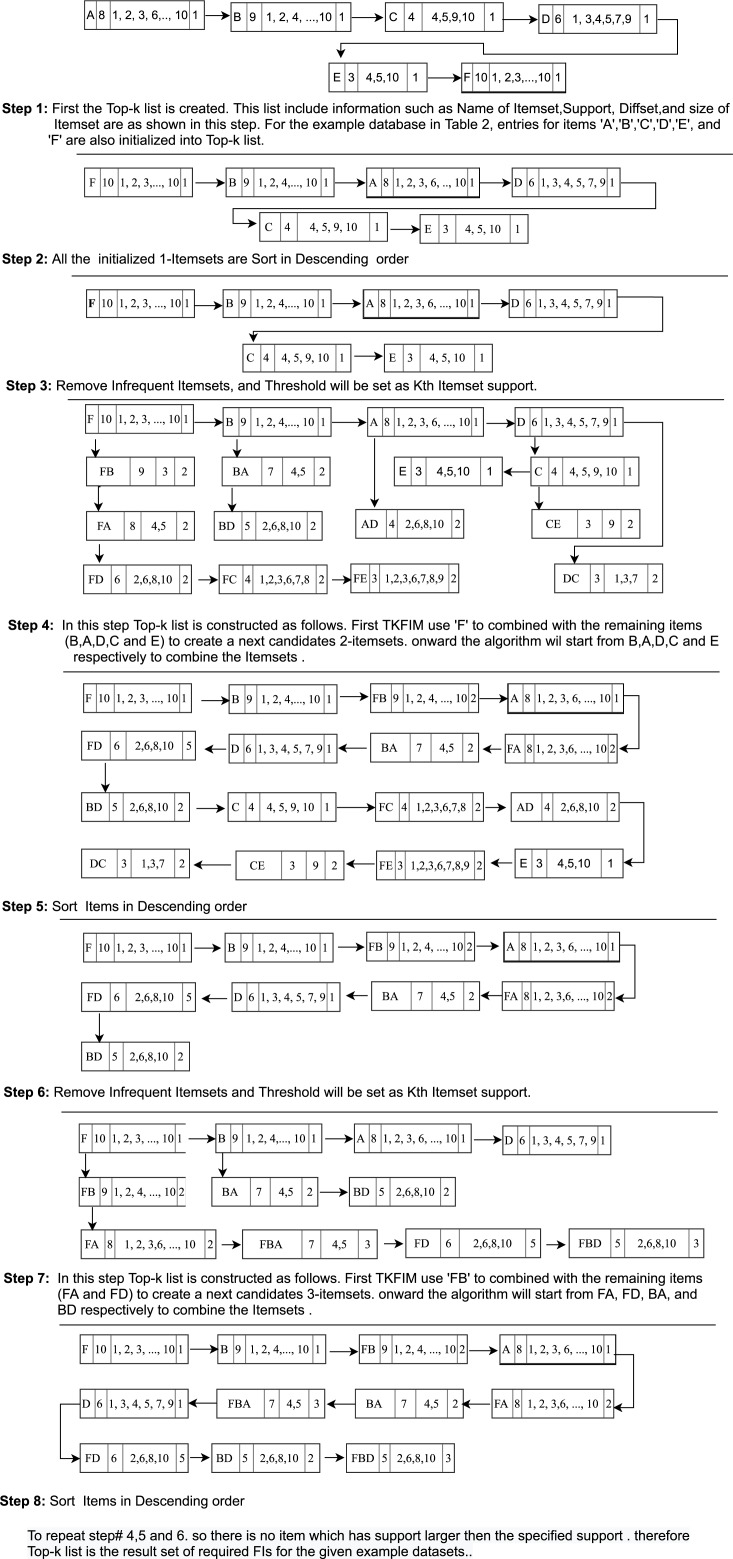
Generation of Top-k list structure.

All items are sorted in descending order, as shown in Fig. 2 (Step 2).

**Table 5:** Vertical database format.

Itemsets	TID
F	1,2,3,4,5,6,7,8,9,10
B	1,2,4,5,6,7,8,9,10
A	1,2,3,6,7,8,9,10
D	1,3,4,5,7,9
C	4,5,9,10
E	4,5,10

### Step 3: Computing 2-Itemsets from 1-Itemsets using Diffsets

#### Pruning process

The candidate’s generation process is based on two constraints. First, it requires the size of the itemsets to be the same. Secondly, both itemset must have the same prefix, which means that each item of the itemset is identical except the last. When both itemsets meet these limitations, k + 1 itemset is generated from k-itemsets with the help of equivalence classes, i.e., 2-itemsets at the next level are generated by joining every two k-itemset. Top-k frequently mined itemsets calculate the difference between the Tids lists in both items to find the support value. This will be the support of the newly generated itemsets. If the new itemset support is higher than the minimum kth support item in the Top-k list, the newly generated itemset is entered in the Top-k list. Likewise, the kth pattern is removed from the list of Top-k list and is designated as infrequent itemsets. After this step, our first iteration is completed, and as a result, each 2-itemsets is generated in the same manner as like itemset ‘FB’. We get the 2-Itemsets, as shown in Fig. 2, Step 4).

**Table 6:** Converted vertical database format.

Itemsets	TID
A	1,2,3,6,7,8,9,10
B	1,2,4,5,6,7,8,9,10
C	4,5,9,10
D	1,3,4,5,7,9
E	4,5,10
F	1,2,3,4,5,6,7,8,9,10

#### Pruning Process in Step 3

As shown in Fig. 2 (Step 1), we compute 2-itemsets from 1-itemsets using difference sets in the given running example. Item ‘F’ and ‘B’ are combined and append together to generate the itemsets ‘FB’ is a Top-k frequent itemset.

The difference set of ‘FB’ and support is calculated, which is 9. The support of ‘FB’ is greater than the minimum support inferred as support of the kth itemset in the Top-k list. Which can be used as support to prune the itemset, then the itemset ‘FB’ is inserted into the Top-k list, and as a result, each 2-itemset are generated in the same manner as like itemset ‘FB,’ as shown in Fig. 2 (Step 3).

### Step 4: Remove Infrequent Itemsets by counting k value

The 1-itemset node *E* and *C* are removed by counting the *k* value in the Top-k list. The 2-itemset nodes are removed because their frequency is less than the minimum kth support item in the Top-k list.

### Step 5: Repeating step-2 to step-4

We start the second iteration by sorting the generated 2-itemset given in Fig. 2 (step 6). We do not need to scan the database any more to link 2-itemset in the Top-k list.

A new entry is created at every occurrence of an item in the Top-k list. The itemsets are initialized with support and Tids-list. The least support itemset used for the pruning process becomes the k itemset in the Top-k list. Then, the itemsets in the list are updated. The Top-k list has been arranged in the order of descent. Finally, all items with less support than the kth item in the Top-k list are removed from the Top-k list, according to the Top-k counting procedure, when the user sets the k value.

The itemset generation process is repeated. So in this iteration of the loop, we compute 3-itemsets from the 2-itemsets using diffset till the least support itemset in the Top-k list. Next level itemsets are generated with the help of equivalence classes. The generated 3-Itemsets are as shown in Fig. 2 (step 7). The process repeats until there are no frequent itemsets found. After that Top-k list is returned as required by the user.

### Step 6: Top-k list is returned

**Figure 3:**
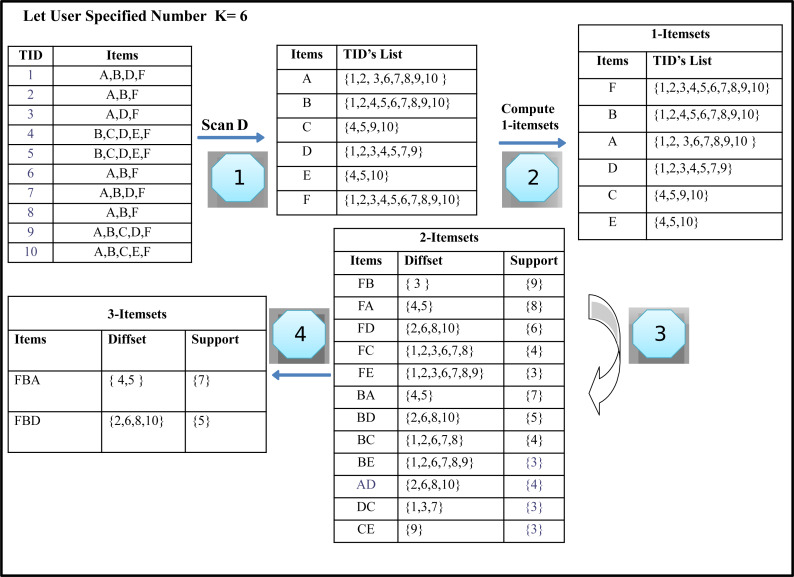
Generation of frequent item sets.

The generated Top-k-List is shown in Fig. 2 (step 8) returned by the TKFIM algorithm. The procedure of the Top-k frequent itemsets mining (TKFIM) method for the example mentioned above is shown in Fig. 3.

## Experimental Results

In this section, we present the evaluation of the proposed system. First, there are standard data sets freely available, so they have been used to evaluate the proposed algorithm. We also compare our algorithms with two recent related methods: the Top-k miner algorithm, which find the Top-k frequent itemsets by using the Candidate Itemset Search (CIS-tree) (Saif-Ur-Rehman et al., 2016) and the BOMO algorithm calculating the common Top-k itemsets (Cheung & Fu, 2004). The fact that they are newly proposed techniques and provided better results than their previous ones is the reason for selecting these two approaches. The techniques calculate frequent element sets by requesting k from the user. The experimental setup and dataset are provided in the following section.

### Experimental setup and datasets

Several experiments were conducted to measure the performance of the algorithm and compare its results with the methods referred to above. The datasets are available freely and downloaded from the Frequent Implementation of Items and Mining (FIMI) data repository (Goethals, 2003). The dataset details are given in Table 7.

The first column of the table shows the dataset name. In the second column, the total number of transactions in a particular data set is described. Whereas the number of items in one transaction is displayed in columns such as *Avg-length* and *Items* column represents the total number of unique elements within a dataset. The last column shows the dataset type. The transaction dataset Chess, Mushroom, Connect, and the T10I4D100 K dataset are three real transactions. Chess is the first real dataset with 75 items and 3,196 dealings. It is a dense dataset with long and short itemset. There are 120 items in the Mushroom dataset and 8124 transactions. The transaction size is an average of 23. This is a sparse dataset that contains a small number of common items. Over 130 items are built to connect. The longest average transaction volume 43 is a dense dataset. T10I4D100K is a synthetic dataset containing 1,000 items and a total transaction count of 100,000 items. This dataset has an average of 10 transactions. The proposed technology was carried out in the programming language version 3.8.1 of Python for experimentation. The Core2duo machine with two gigabytes of memory and Windows 10 operating system is the experimental environment.

**Table 7:** Datasets characteristics.

.**Datasets**	**# Transactions**	**Ave-length**	**#Items**	**Type**	**Data set source**
Chess	3,196	37	76	Real	http://fimi.uantwerpen.be/data/chess.dat
Mushroom	8124	23	120	Real	http://fimi.uantwerpen.be/data/mushroom.dat
Connect	67,557	43	130	Real	http://fimi.uantwerpen.be/data/connect.dat
T10I4D100K	100,000	10	1,000	Synthetic	http://fimi.uantwerpen.be/data/T40I10D100K.dat

### Results

We have performed experiments on three real and one synthetic dataset. The first experiment has been performed on the aforementioned dataset and the results of the TKFIM as shown in Table 8. The first column of the Table shows the transaction dataset sequence, the second column contains the data set name, the third column describes the TKFIM data of *k* = 10, the fourth column shows the transaction datasets of Top-k miners, and the second column shows the results of the BOMO procedure and the last column shows the itemsets missing by BOMO. In the first stage, we compare the frequent itemsets generated by TKFIM with the Top-k miner and BOMO approach. For the Top-10 frequent itemsets on four datasets, we show the results in Table 8.

In the past, the Top-N and the Top-N support threshold for frequently used item sets are mining methods, such as FP-growth, Top-N, and CRMN.

These methods fail to provide important support patterns due to the tuning of the threshold parameters. The BOMO is one of the top pattern mining approaches using two parameters (Cheung & Fu, 2004). The N-most interesting itemset is the union of the N-most interesting itemsets with the highest supports for the value of some k.

To demonstrate BOMO with specified parameters k, having considered the same values for the specified parameter, we compute Top-9 frequent itemsets on all datasets as shown in Table 8. The BOMO procedure returns the wrong result and misses essential patterns. The value of the k input parameter is taken up by our proposed TKFIM and mine all Top-k Items in the dataset and does not miss the highest support pattern in the result set, as it is clear from Table 8. The proposed system’s performance is now a matter of question, so we present its result in the next section.

### Performance results of TKFIM

**Figure 4:**
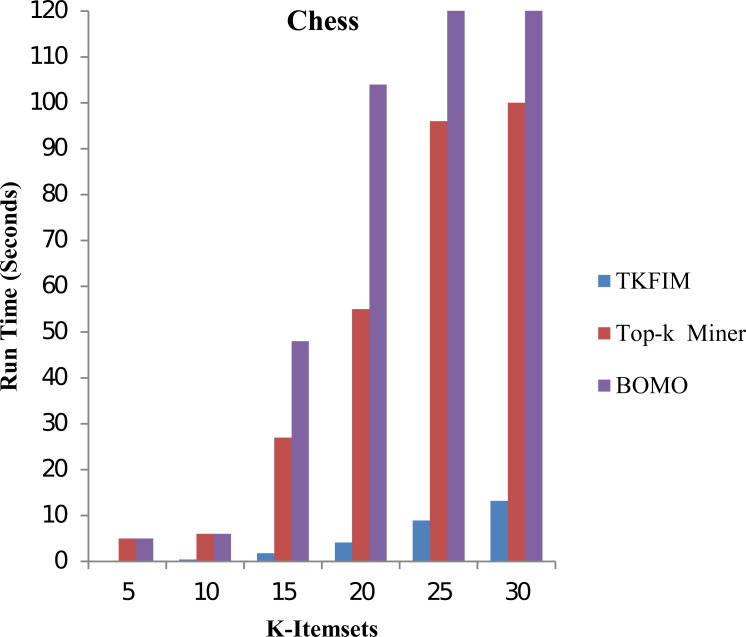
Performance results of TKFIM on chess datasets.

In the second phase, we study our proposed TKFIM method’s performance without support threshold parameter performing sets of experiments on each dataset mentioned above, shown in Table 7. To conduct experiments, the input value of k, provided by the user, is set in the range of five to thirty. Figure 4 shows the performance results of all the approaches mentioned above on the “chess” dataset.

In this figure, each algorithm has its execution time on the vertical side, while each algorithm performance is shown with different k-values on the horizontal bar. The Top-k miner performs poorly in a large value of k provided by the user. BOMO produces a large number of infrequent patterns that increase its run time. In all cases, with a k value between five and thirty, the BOMO algorithm has shown a poor performance. The blue bar represents the proposed algorithm TKFIM, the brown bar expresses the Top-k miner results, and the purple bar describes the BOMO algorithm results. The presented results illustrate that TKFIM outperforms both the Top-k miner and BOMO procedures. As shown in the graph, the TKFIM discovers the top five frequent items in 0.03 s, where the Top-k and BOMO consume the same amount of time to do the same job.

**Table 8:** Top-k FIs mined by Top-k miner, BOMO and TKFIM.

S.No	Datasets	Results of TKFIM for k= 10	Results of Top-k Miner For k= 10	Results of BOMO for *N* = 3, k=3	Wrong results itemsets missed by BOMO
**1.**	Chess	1) 58=3195	1) 58=3195	1) 58= 3195	40= 3170
		2) 52=3185	2) 52=3185	2) 52= 3185	58,40= 3169
		3) 58,52=3184	3) 58,52=3184	3) 29 = 3181	52,40=3159
		4) 29 = 3181	4) 29 = 3181	4) 58,52 = 3184	
		5) 58,29=3180	5) 58,29=3180	5) 58,29 = 3180	
		6) 40=3170, 52,29=3170	6) 40=3170, 52,29=3170	6) 52,29= 3170	
		7) 58,52,29=3169 58,40=3169	7) 58,52,29=3169 58,40=3169	7) 58,52,29=3169	
		8) 40=3159	8) 40=3159	8) 58,52,40=3158	
		9) 58,52,40=3158	9) 58,52,40=3158	9) 58,29,40=3154	
		10)29,40= 3155	10)29,40= 3155		
**2.**	Mushroom	1) 85=8124	1) 85=8124	1) 85 = 8124	1) 90 = 7488
		2) 86=7924 85,86=7924	2) 86=7924 85,86=7924	2) 86 = 7924	2) 85,90 = 7488
		3) 34=7914 34,85=7914	3) 34=7914 34,85=7914	3) 34 = 7914	3) 34 = 7914
		4) 34,86=7906 34,85,86=7906	4) 34,86=7906 34,85,86=7906	4) 85,86 = 7924	5)85,86,34,90=7906
		5) 90=7488 90,85=7488	5) 90=7488 90,85=7488	5) 85,34 = 7914	6) 86,34,90=7906
		6) 90,34=7296 90,85,34=7296	6) 90,34=7296 90,85,34=7296	6) 86,34 =7906	7) 34,90 = 7296
		7) 90,86=7288 90,34,86=7288 90,85,85=7288 90,85,34,86=7288	7) 90,86=7288 90,34,86=7288 90,85,85=7288 90,85,34,86=7288	7) 85,86,34 =7906	8) 86,90=7288
		8) 36=6812 36,85=6812	8) 36=6812 36,85=6812	8) 34,85,90 = 7296	
		9) 36,86 = 6620 36,85,86=6620	9) 36,86 = 6620 36,85,86=6620	9) 85,86,90 = 7288	
		10) 36,34=6602 36, 86, 34=6602 36, 85, 34=6602 36 85 86 34 =6602	10) 36,34=6602 36, 86, 34=6602 36, 85, 34=6602 36 85 86 34 =6602		
**3.**	Connect	1) 91 = 67473	1) 91 = 67473	1) 91=67473	1) 75= 67245
		2) 109=67469	2) 109=67469	2) 109=67469	2) 91, 75= 67161
		3) 127= 67465	3) 127= 67465	3) 127= 67465	3) 109,75=67157
		4) 91,109=67385	4) 91,109=67385	4) 91,109 = 67385	
		5) 91,127=67381	5) 91,127=67381	5) 91, 127=67381	
		6) 109,127=67377	6) 109,127=67377	6) 109,127=67377	
		7) 91,109,127=6729	7) 91,109,127=67293	7) 91,109,127=67293	
		8) 75=67245	8) 75=67245	8) 91,109,75=67073	
		9) 91,75=67161	9) 91,75=67161	9) 91,127,75=67069	
		10) 109,75=67157	10) 109,75=67157		
**4.**	T10I4D100K	1) 368 = 28738	1) 368 = 28738	1) 368 = 28738	1) 510 = 20125
		2) 529 = 23384	2) 529 = 23384	2) 529 = 23384	2) 419 = 20216
		3) 829 = 23121	3) 829 = 23121	3) 829 = 23121	3) 217 = 19326
		4) 419 = 20216	4) 510 = 20125	4) 368,529 =7500	4) 489 = 18921
		5) 510 = 20125	5) 419 = 20216	5) 368,829 = 6957	5) 682= 17427
		6) 217 = 19326	6) 217 = 19326	6) 368,682 =6130	6) 914 = 17343
		7) 489 = 18921	7) 489 = 18921	7) 682,368,489 =2420	7) 692= 17203
		8) 682= 17427	8) 682= 17427	8) 368,529,692 = 2373	
		9) 914 = 17343	9) 914 = 17343	9) 368,529,829 = 2288	
		10) 692= 17203	10) 692= 17203		

Similarly, we discovered that the top ten frequent itemsets by TKFIM take 0.4 s, whereas the Top-k and BOMO take six seconds to do the same job. The top thirty frequent itemsets by TKFIM take 13.21 s, whereas the Top-k consume a hundred seconds, and BOMO takes 1,010 s. Our proposed technique’s performance gain over BOMO and Top-k miner on Chess dataset are 97.14 and 92.70 percent, respectively.

**Figure 5:**
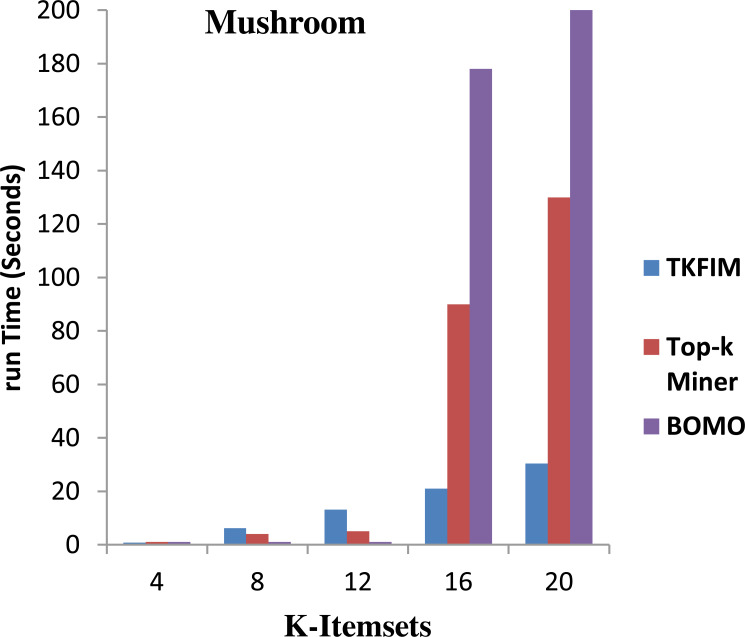
Performance results of TKFIM on mushroom dataset.

Figure 5 shows the results of performance on the Mushroom dataset. The algorithm’s execution time on the vertical side is shown in this figure, where the horizontal bar reflects every algorithm’s performance for different values of k. The Top-k miner performs well on smaller values of k provided by the user. However, BOMO poorly and increase its run time. In all cases of k between four and twenty, the BOMO algorithm has shown a poor performance.

Further, the blue bar represents the proposed algorithm, the brown bar expresses the Top-k miner results, and the purple bar describes the BOMO algorithm results. The results show that the performance for a small amount of k is degraded compared to Top-k miner because the data set contains only a few short, frequently used items.

The TKFIM discovers the top four common items in 0.74 s, in which the Top-k and BOMO take a second to do the same task, as shown in the graph. Similarly, TKFIM found that the top eight frequent itemsets take 1.24 s, and BOMO took four seconds to do the same job. It also found that TKFIM for finding top-20 frequent itemset takes 30.34 s, while the Top-k miner takes 130 s and BOMO takes 1,010 s.

The performance gain of our proposed technique over BOMO and Top-k miner on Mushroom dataset is 100 and 35.87 percent, respectively.

**Figure 6:**
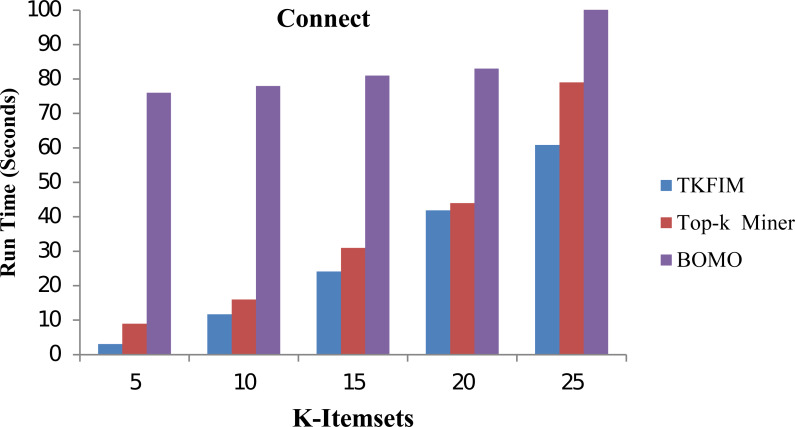
Performance results of TKFIM on connect dataset.

In Fig. 6, the performance results for the Connect dataset are shown. In this figure, each algorithm has its execution time on the vertical side of this figure, while each algorithm performance with different k-values is shown on the horizontal bar. The Top-k miner performs better on small values of k provided by the user. However, BOMO suffered from increased run time. In all cases of k between 5 and 25,n the BOMO algorithm shown a poor performance.

Further, the blue bar represents the proposed algorithm, the brown bar expresses the Top-k miner results, and the purple bar describes the BOMO algorithm results. As shown in the graph, the TKFIM discovers the top five frequent items in 3.07 s, the Top-k miner takes nine seconds, and BOMO produces the result of the top five frequent items in 76 s. Similarly, to find the top 25 frequent itemsets by TKFIM took 60.87 s, Top-k miner took 79 s, and BOMO consumed 597 s. The performance gain of our proposed technique over BOMO and Top-k miner on Connect dataset is 78.10 and 28.53 percent, respectively.

**Figure 7:**
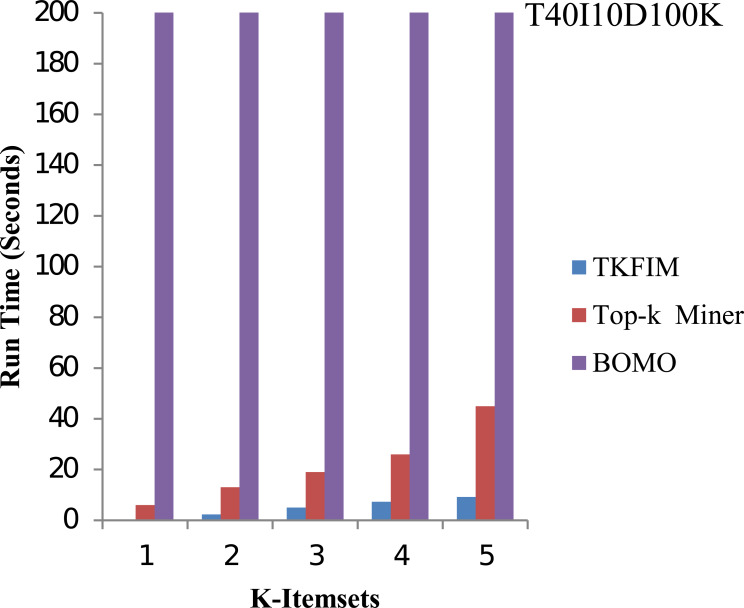
Performance results of TKFIM on synthetic dataset.

The results of synthetic dataset T40I10D100K are shown in Fig. 7. The pattern length is short in synthetic datasets, but it has 1,000 items. In this figure, the execution time of each method is shown on the vertical side, whereas the horizontal bar represents the performance of each technique for varying values of k. The Top-k miner performs better on small amounts of k provided by the user. We performed experiments for k between one and five.

Further, the blue bar represents the proposed algorithm, the brown bar expresses the Top-k miner results, and the purple bar describes the BOMO algorithm results. When frequent patterns of a higher length are discovered, BOMO starts to suffer from a radical increase in execution time. As shown in the graph, the TKFIM identifies the top one frequent item in 0.06 s, the Top-k miner takes six seconds, and BOMO produces a result of the top one frequent items in 76 s. Similarly, to find the top five frequent itemsets, TKFIM takes 9.18 s, Top-k miner takes 45 s, and BOMO consumes 1,699 s. Our proposed technique’s performance gain over BOMO and Top-k miner on the T1014D100K dataset is 99.70 and 81.27 percent, respectively.

### Memory usage of TKFIM

**Figure 8:**
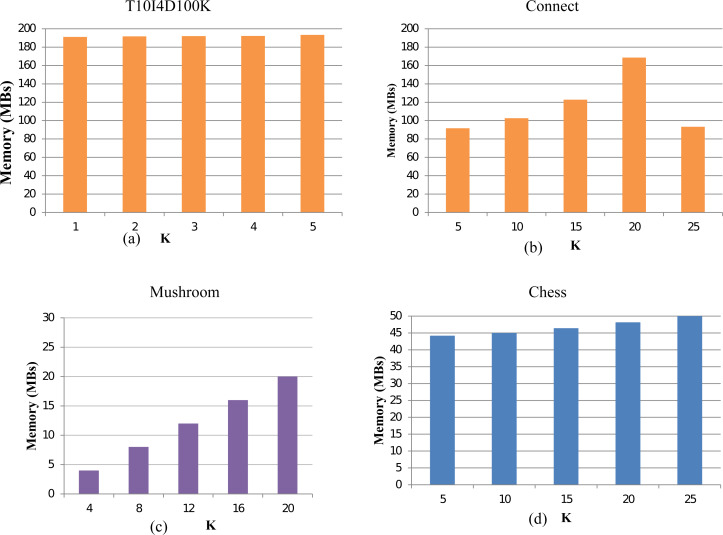
Memory usage of TKFIM algorithm.

To evaluate the memory consumption of the TKFIM algorithms, we execute our method for all the datasets. For the Chess dataset, TKFIM consumes a 1MB memory to obtain the result of top-5, top-10, and top-15 FIs. However, it takes 2 MB memory to produced top-25 and top-30 itemsets. Similarly, on mushroom datasets, it consumes 2MB to compute top-16 FIs and top-20 FIs. In all experiments, the gap of memory consumption increases by 1MB while increasing the value of k. In conclusion, the TKFIM algorithm presents the least memory for all the experimental datasets for a lesser value of K. The results of the various dataset are shown in Fig. 8. The memory consumption results of dataset T1014D100K, Connect, Mushroom, and Chess are shown in Figs. 8A, 8B, 8C, and 8D respecitively. These results were computed with the help of code which may not very accurate as because of different factors involment in memory consumpution. However, one thing which is apperent from these results is that less memory is needed for lesser value of K.

## Conclusion

Frequent itemset mining is an exciting branch of data mining that focuses on looking at frequently co-occurring items. The items could be from patterns in any dataset like a market basket, word usage in documents, clicking behavior of users, gene sequencing, etc. Due to its wide range of applications, researchers are always trying to produce effective solutions. This research has also proposed, designed, and developed an algorithm based on equivalence classes and diffset theory for mining Top-k frequent itemsets. It is found that the TKFIM has outperformed the results of these approaches in terms of execution and performance, achieving 92.70, 35.87, 28.53, and 81.27 percent gain on Top-k miner using Chess, Mushroom, Connect, and T1014D100K datasets, respectively.

Similarly, TKFIM has achieved 97.14, 100, 78.10, and 99.70 percent on BOMO using Chess, Mushroom, Connect, and T1014D100K datasets, respectively. The proposed TKFIM technique outperforms its counterpart on every dataset. In the future, we plan to adapt this work to solve other data mining tasks like sequential pattern mining, temporal FIs mining, FIs based clustering, and incremental frequent itemsets mining.

##  Supplemental Information

10.7717/peerj-cs.385/supp-1 Supplemental Information 1 Code used to implement the project

10.7717/peerj-cs.385/supp-2 Supplemental Information 2 Dataset used in this research

10.7717/peerj-cs.385/supp-3 Supplemental Information 3 Sources of datasets used

## Additional Information and Declarations

### Data Availability

The following information was supplied regarding data availability: The data set and code are available in the Supplemental Files. Data was taken from the Frequent Itemset Mining Dataset Repository (http://fimi.uantwerpen.be/data/): - T10I4D100K: http://fimi.uantwerpen.be/data/T40I10D100K.dat
- Chess: http://fimi.uantwerpen.be/data/chess.dat
- Connect: http://fimi.uantwerpen.be/data/connect.dat
- Mushroom: http://fimi.uantwerpen.be/data/mushroom.dat.
