# Supplementary material for: TKFIM: Top-K frequent itemset mining technique based on equivalence classes
Source: PeerJ Comput Sci. 2021 Mar 8;7:e385. doi: 10.7717/peerj-cs.385 (PMC7959650; doi:10.7717/peerj-cs.385)
Supplement: Supplemental Information 3 [file peerj-cs-07-385-s003.docx]

**Frequent Itemset Mining Dataset Repository**

**T10I4D100K**

<http://fimi.uantwerpen.be/data/T40I10D100K.dat>

**Chess**

<http://fimi.uantwerpen.be/data/chess.dat>

**Connect**

<http://fimi.uantwerpen.be/data/connect.dat>

**Mushroom**

<http://fimi.uantwerpen.be/data/mushroom.dat>
